# Interpreting the effects of altered brain anatomical connectivity on fMRI functional connectivity: a role for computational neural modeling

**DOI:** 10.3389/fnhum.2013.00649

**Published:** 2013-11-11

**Authors:** Barry Horwitz, Chuhern Hwang, Jeff Alstott

**Affiliations:** ^1^Brain Imaging and Modeling Section, National Institute on Deafness and Other Communication Disorders, National Institutes of HealthBethesda, MD, USA; ^2^National Institute of Biomedical Imaging and Bioengineering, National Institutes of HealthBethesda, MD, USA; ^3^Department of Biomedical Engineering, University of VirginiaCharlottesville, VA, USA; ^4^Section on Critical Brain Dynamics, National Institute of Mental Health, National Institutes of HealthBethesda, MD, USA; ^5^Brain Mapping Unit, Behavioural and Clinical Neuroscience Institute, University of CambridgeCambridgeshire, UK

**Keywords:** neural modeling, fMRI, functional connectivity, brain disorders, human brain

## Abstract

Recently, there have been a large number of studies using resting state fMRI to characterize abnormal brain connectivity in patients with a variety of neurological, psychiatric, and developmental disorders. However, interpreting what the differences in resting state fMRI functional connectivity (rsfMRI-FC) actually reflect in terms of the underlying neural pathology has proved to be elusive because of the complexity of brain anatomical connectivity. The same is the case for task-based fMRI studies. In the last few years, several groups have used large-scale neural modeling to help provide some insight into the relationship between brain anatomical connectivity and the corresponding patterns of fMRI-FC. In this paper we review several efforts at using large-scale neural modeling to investigate the relationship between structural connectivity and functional/effective connectivity to determine how alterations in structural connectivity are manifested in altered patterns of functional/effective connectivity. Because the alterations made in the anatomical connectivity between specific brain regions in the model are known in detail, one can use the results of these simulations to determine the corresponding alterations in rsfMRI-FC. Many of these simulation studies found that structural connectivity changes do not necessarily result in matching changes in functional/effective connectivity in the areas of structural modification. Often, it was observed that increases in functional/effective connectivity in the altered brain did not necessarily correspond to increases in the strength of the anatomical connection weights. Note that increases in rsfMRI-FC in patients have been interpreted in some cases as resulting from neural plasticity. These results suggest that this interpretation can be mistaken. The relevance of these simulation findings to the use of functional/effective fMRI connectivity as biomarkers for brain disorders is also discussed.

## Introduction

In the past few years, brain connectivity analyses have become important tools in the investigation of brain disorders [besides the articles in this Special Issue, see, for example, the Frontiers in Systems Neuroscience Special Issue on Brain Connectivity Analysis: Investigating Brain Disorders (Horovitz and Horwitz, 2012; Horwitz and Horovitz, 2012)]^1^. Probably the most common connectivity studies have used diffusion tensor imaging (DTI)^2^ to investigate brain anatomical connectivity and functional magnetic resonance imaging (fMRI) to examine functional and/or effective connectivity. Although there still exists some confusion in the literature as to the definition of the latter two terms (Horwitz, 2003), for the purposes of this article we follow Friston (1994) and take functional connectivity to denote a statistical relationship between the functional neuroimaging signals in two or more brain regions (e.g., a correlation coefficient or a regression coefficient), and effective connectivity to mean the direct effect of one brain region's activity on another during a specified experimental condition (e.g., the functional strength of the directed anatomical link from one region to another during a particular task).

The earliest functional connectivity neuroimaging studies that used positron emission tomographic (PET) data were acquired during the so-called resting state (e.g., Horwitz et al., 1984), but gave way a few years later to task-based studies (Horwitz et al., 1992), especially when fMRI became available (e.g., Friston et al., 1997; Bokde et al., 2001). Thus, there developed a substantial literature on activation studies of patients with brain disorders employing functional/effective brain connectivity analysis methods (e.g., Horwitz et al., 1995; Bokde et al., 2006; Just et al., 2007; Rytsar et al., 2011). However, during the past decade or so, there has been an explosion in the number of studies using resting state fMRI (rsfMRI) to characterize functional brain connectivity in normal subjects (e.g., Biswal et al., 1995; Yeo et al., 2011) and in patients with a variety of neurological, psychiatric, and developmental disorders (e.g., Cherkassky et al., 2006; Wang et al., 2006; Alexander-Bloch et al., 2010; Lynall et al., 2010; Damoiseaux et al., 2012; Venkataraman et al., 2012; Lynch et al., 2013). The literature on functional neuroimaging connectivity studies in brain disorder patients is now huge, and obviously difficult to summarize. It is possible to generalize, however, and say that almost all published studies have found differences in functional (or effective) connectivity between patients and healthy control subjects. Often, the differences correspond to a decreased connectivity in the patients, although in many instances, increased interregional connectivity has been reported; sometimes, both types of differences are found together (e.g., Horwitz et al., 1995; Damoiseaux et al., 2012; Venkataraman et al., 2012). Note also that it has become widely appreciated that neuroimaging studies of brain connectivity, both functional and structural, have the potential for generating useful biomarkers for the detection and diagnosis of brain disorders and for the assessment of their treatment [for example, for Alzheimer's disease (AD), see (Horwitz and Rowe, 2011; Damoiseaux, 2012)].

Nonetheless, the question does arise as to how these alterations in functional/effective connectivity should be interpreted. For example, some researchers have suggested that an increased functional/effective connectivity may reflect some type of compensatory change that helps maintain normal function in spite of aberrant function in other parts of the brain. Also, can one attribute, as is often done, a reduced functional/effective connectivity to a decreased structural link between two brain regions? A decreased structural link may manifest itself as a reduced axonal input (either fewer axons or less effective synaptic inputs) from one neural population to another. How can we determine if these interpretations of functional brain connectivity analyses are justified? With respect to human brain disorders, it is obviously hard (indeed impossible at present) to actually do this using experimental data, since invasive techniques cannot be employed. Furthermore, the complexity of the mammalian brain mostly precludes any sort of direct comparison between measures of interregional neuronal connectivity and fMRI based measures in non-human animals, although some recent efforts in this direction (Logothetis, 2012), including using optogenetic approaches (Lee, 2011), show some promise. Rather, these issues have started to be addressed using computational neural modeling.

In this paper, we will discuss a few of these neural modeling efforts in the section entitled Simulated fMRI Data and Functional/Effective Connectivity, focusing especially on what has been learned about how to interpret differences in functional/effective connectivity between patients and healthy subjects in Simulating the Effect of Altered Anatomical Connectivity on Functional/Effective Connectivity. We will conclude in The Role of Simulation in the Development of fMRI Biomarkers with some thoughts on the role that neural modeling can play in developing fMRI functional/effective connectivity based biomarkers for various aspects related to the detection and treatment of brain disorders.

## Simulated fMRI data and functional/effective connectivity

There have been a number of investigators who have developed multi-region network models that can simulate functional neuroimaging data. These models vary with respect to how “biologically realistic” are the elements that comprise each model. Efforts of this sort that deal with the kind of task-related flow/metabolic neuroimaging data generated by PET and fMRI began in the mid-to-late 90s (Arbib et al., 1995; Tagamets and Horwitz, 1998; Horwitz and Tagamets, 1999), and have increased dramatically since then (e.g., Corchs and Deco, 2004; Deco et al., 2004, 2008; Husain et al., 2004; Edin et al., 2007; Marreiros et al., 2008; Smith et al., 2013). Recently, a number of groups have developed modeling platforms for examining simulated rsfMRI data (for instance, Alstott et al., 2009; Honey et al., 2009; Cabral et al., 2011, 2012b; Smith et al., 2011; Ritter et al., 2013). Although some of these modeling efforts have focused on examining differences between healthy subjects and patients, others have used the computational models to address how specific tasks are implemented at the neural level. Relevant to the discussion that will follow, we will illustrate three of these modeling efforts.

**Figure 1:**
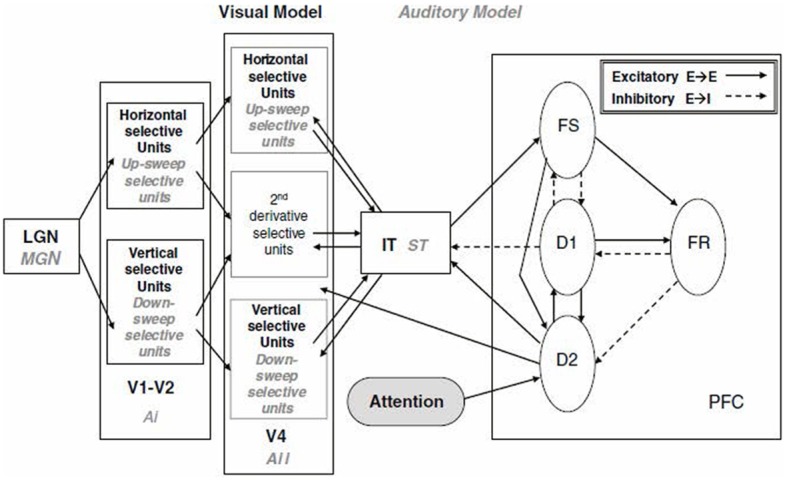
**Large-scale neural network models of the visual and auditory object processing pathways (Tagamets and Horwitz, 1998; Husain et al., 2004)**. Shown are the modules specific to the visual model (LGN, V1-V2, V4, IT) in black-bold and those specific to the corresponding auditory model (MGN, Ai, Aii, ST, PFC) in gray-italics. Within each module are sub-modules. The PFC module is common to both models and shown are its sub-modules. Each sub-module contains 81 basic neural elements consisting of an interacting pair of excitatory and inhibitory units (Wilson and Cowan, 1972). Connections between modules are display (solid: excitatory-to-excitatory; dashed: excitatory to inhibitory). Models perform a delayed match-to-sample task for either visual objects (combinations of horizontal and vertical lines) or auditory objects (combinations of pure tones and up- and down-frequency sweeps. Abbreviations: LGN, lateral geniculate nucleus; MGN, medial geniculate nucleus; V1-V2, primary and secondary visual cortex; V4, extrastriate visual cortex; IT, inferior-temporal cortex; Ai, primary auditory cortex; Aii, secondary auditory cortex; ST, superior temporal gyrus-sulcus; PFC, prefrontal cortex. Taken from Horwitz and Smith (2008).

The model developed by Tagamets and Horwitz (1998), although initially applied to regional cerebral blood flow (rCBF) PET data, was soon extended to blood oxygenation level dependent (BOLD) fMRI (Horwitz and Tagamets, 1999). The model was designed to simulate a short-term memory task for visual objects. It consisted of a number of distinct neuronal populations along the ventral visual processing stream arranged in the following brain regions (see Figure 1): primary and secondary visual cortex (V1), extrastriate visual cortex (V4 and IT), and prefrontal cortex (PFC). The visual feature that was modeled was object shape, and thus the V1 neurons were configured to respond to line orientation (for simplicity, the orientations were restricted to horizontal and vertical). The basic neuronal element in each module was a modified Wilson–Cowan unit (Wilson and Cowan, 1972), which consists of an excitatory-inhibitory pair that can be thought of as representing an extremely simplified cortical column. Each model population contained 81 basic elements. The populations were connected together based, as much as possible, on known primate neuroanatomy. For example, connectivity was such that the spatial receptive field increased as one moved down the object processing pathway. The PFC region contained four distinct simulated neuronal populations whose activities were designed to correspond to the experimental data of Funahashi et al. (1990), obtained from monkeys during the performance of a delayed response task. The model simulated a delayed match-to-sample task, in which a simulated object is presented for a short period of time, there is a delay period, and a second object is presented. The goal was to determine if the second object was the same as the first. An intertrial interval then occurred, and another trial began. The entire simulation corresponded to multiple trials, as would occur during an actual PET or fMRI study. The properties of the simulated neurons were configured so that their firing patterns were similar to those obtained from electrophysiological monkey studies. The spatiotemporal integrated synaptic activities (absolute value of the excitatory and inhibitory neuronal inputs) were assumed to represent the rCBF in each area for PET (Tagamets and Horwitz, 1998). For fMRI, the integrated synaptic activities were calculated for a time period of about 50 ms (the time needed to acquire a single MRI slice), convolved with a function representing the hemodynamic response, and then downsampled each TR (e.g., *TR* = 2 s) to represent simulated BOLD-fMRI (Horwitz and Tagamets, 1999). Good agreement was obtained between the simulated PET data and the experimental PET data of Haxby et al. (1995) (see Tagamets and Horwitz, 1998 for details). This model was later modified by Husain et al. (2004) to produce a simulation model for auditory object processing. Both the visual and auditory models were subsequently employed to simulate fMRI-functional connectivity data (time-series correlations) (Horwitz et al., 2005; Kim and Horwitz, 2008).

It is important to notice that these kinds of multiregion large-scale simulations require a combination of three component models. The first component is a structural model that indicates how the simulated brain regions are anatomically linked, and what are the strengths of the linkages. The second component is a neuronal model. The third component is a hemodynamic response model that converts the neural activity into a neuroimaging signal. In the simulations just discussed, the structural model was based on primate neuroanatomy, the neuronal model was the Wilson-Cowan unit, and the hemodynamic model was a simple Poisson convolution function acting on the integrated synaptic activity.

**Figure 2:**
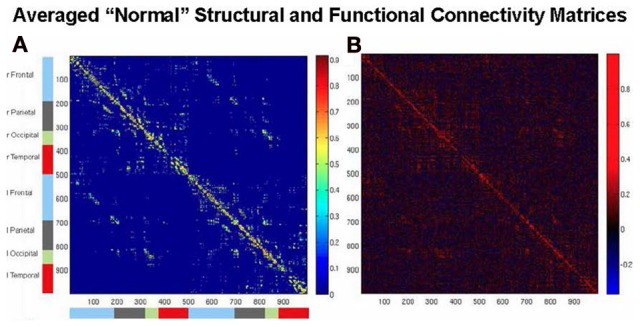
**Brain connectivity matrices. (A)** Structural connectivity matrix among the set of 998 ROIs of the average of the DSI data of five normal subjects of Hagmann et al. (2008). **(B)** Functional connectivity matrix of Pearson correlations from the computational model used by Honey et al. (2009) and Alstott et al. (2009) for the averaged structural matrix of **(A)**, showing relatively high simulated rsfMRI-FC within lobes, and lower rsfMRI-FC between hemispheres.

An example of simulating human rsfMRI data was provided by Honey et al. (2009). They used a structural model based on diffusion spectrum imaging (DSI) data obtained from five normal human participants originally described by Hagmann et al. (2008) (see Figure 2A)^3^. The structural connections were evaluated from streamline tractography values between each pair of 998 cortical regions. The neural model assigned to each of these regions employed the neural mass model of Breakspear et al. (2003), which represents an ensemble of excitatory and inhibitory neurons possessing both ligand-gated and voltage-gated membrane channels. A non-linear hemodynamic model was used to convert simulated neural activity into simulated BOLD fMRI data (Friston et al., 2000) (see Figure 2B). Honey et al. (2009) used this formulation to compare simulated rsfMRI data against actual fMRI data obtained in the same subjects from whom the DSI data were acquired. Their main conclusion was that in both the simulated and experimental data, the underlying structural connectivity constrained the pattern of resting state functional connectivity, although some functional connectivity between non-anatomically connected regions was also present. These findings were supported by a resting state fMRI functional connectivity (rsfMRI-FC) study in monkey by Adachi et al. (2012), who also performed a simulation study employing the modeling framework of an earlier Honey et al. paper (2007).

Gustavo Deco and his colleague have used a comparable modeling approach to that of Honey et al. (2009) to investigate other aspects of rsfMRI data (Deco et al., 2009; Cabral et al., 2011). For instance, Cabral et al. (2011) found that slow power fluctuations in gamma (60 Hz) oscillations at the local neural level could result in long-range interregional resting state synchrony at very low frequencies (<0.1 Hz), indicating that local neural dynamics can have an important effect on network connectivity patterns [see Hlinka and Coombes (2012) for a similar finding]. Cabral and colleagues employed the same structural model as used by Honey et al. [although downsampled to 66 regions of interest (ROIs) from the full set of 998 of the original], as well as the same hemodynamic model. However, they utilized a simpler neural model: the Kuramoto oscillator (Kuramoto, 1984), which has been used extensively to examine the behavior of coupled oscillatory systems. Other component models were employed in other studies by this group. For example, in Deco et al. (2009), the structural model was that of the macaque monkey obtained using anatomical connectivity values from the CoCoMac database (Kotter, 2004), and the neural model utilized the Wilson-Cowan formulation (Wilson and Cowan, 1972). An important insight they found was the critical role that conduction delays between connected brain regions play in allowing synchrony to emerge.

It is worth noting that the main reason different component models are used in different studies is because each study is attempting to understand just a few aspects of the data. So, a neural oscillator model was used when the goal of the study was to relate high frequency neural activity to low frequency BOLD activity, as was the case in the Cabral et al. paper (2011). Some of the other studies that were mentioned placed more emphasis on neural realism, and so models more directly inspired by neurons were employed. In all cases, because there are so many interacting neural units in these large-scale simulations, the simplest neural model that embodied the crucial features of the data was chosen. As more such studies appear in the future, it will be important to determine the degree to which the simulated results depend on the exact nature of the component models that are used. For example, resting state studies may well be somewhat insensitive to the exact neural and metabolic models that are employed, whereas task-based studies may show a strong dependence on the composition of the neural model that is used.

An important issue to mention here is that because these large-scale models can produce multiregional simulated fMRI data that are comparable to experimental data, many of the same analysis techniques that are applied to the experimental data can be applied as well to the simulated data. This is important, given that network analysis techniques, especially graph theory, are commonly employed in MRI studies of structural and functional connectivity (Achard et al., 2006; Bassett and Bullmore, 2006; Bullmore and Sporns, 2009; Sporns, 2012), and as we shall see, these network metrics can be utilized for investigating brain disorders.

Finally, even though the current paper is focused on fMRI functional/effective connectivity, it is worth noting that there also is a vast literature in which brain connectivity analyses are performed on EEG/MEG data (e.g., Gevins and Bressler, 1988; Gross et al., 2001; Daunizeau et al., 2009; Brookes et al., 2011; Rong et al., 2011), and large-scale neural modeling has been employed to help interpret experimental findings (for example, see Wendling et al., 2009; Banerjee et al., 2012).

## Simulating the effect of altered anatomical connectivity on functional/effective connectivity

One important application of these large-scale simulation models has been the investigation of the effects of various types of brain alterations on functional/effective connectivity. As we pointed out in the Introduction, interpreting the results of a brain alteration in real experimental data is difficult because of the complexity of the underlying neural architecture, coupled with neuroplasticity that can occur in real brains subsequent to the alteration. In a large-scale simulation, however, everything is under the control of the researcher, and, in principle, everything that goes on during a simulation can be tracked and evaluated.

Cabral and colleagues published a study that nicely illustrates what can be learned about bran disorders from simulations of rsfMRI (Cabral et al., 2012b). In this investigation, the effects of structural disconnection on rsfMRI-FC was examined using a large-scale neural modeling framework. The structural model that was employed consisted of 90 ROIs derived from DTI data acquired from 21 healthy participants; the neural model for each ROI, based on the dynamical equations of Mattia and Del Giudice (2002), generated spontaneous neural activity; and the hemodynamic model that the authors used was the Balloon-Windkessel model of Friston et al. (2000). The simulated rsfMRI-FC was evaluated as the temporal correlation between ROI time series, and graph theoretic measures (Bassett and Bullmore, 2006; Bullmore and Sporns, 2009) were employed to characterize the pattern of connectivity among all the ROIs. Two types of structural disconnection were simulated—global and local. In the equations relating the change in neural activity (firing rate) in one region (region *n*) to that in other regions, there exists a term *kC_np_*, where *k* is the global excitatory coupling between all regions and *C_np_* is the structural coupling strength from region *p* to region *n*. For the global disconnection simulations, *k* was uniformly reduced. It was found that a number of the graph theoretic metrics changed, resulting in a less globally correlated and globally integrated set of BOLD values. The second kind of structural disconnection that they simulated was a more localized type, in which Cabral and colleagues successively removed randomly 1% of the possible links (what they termed “pruning the matrix”). The results for this case were similar to that for the global disconnection case—a reduction in functional connectivity leading to reduced global integration.

Cabral et al. (2012a) went on to explicitly compare simulated rsfMRI-FC with experimental data acquired from patients with schizophrenia (Lynall et al., 2010). The experimental data showed that, compared to healthy control subjects, the schizophrenia patients had weakened functional connectivity and an increased diversity of functional connections. Cabral and colleagues tested the hypothesis that these disrupted functional networks in the patients could be explained by a global decrease in structural coupling between cortical regions. They found that a small decrease in the global structural coupling parameter, *k*, yielded a reduced functional connectivity that resulted in graph theoretic changes similar to those documented by Lynall et al. (2010).

**Figure 3:**
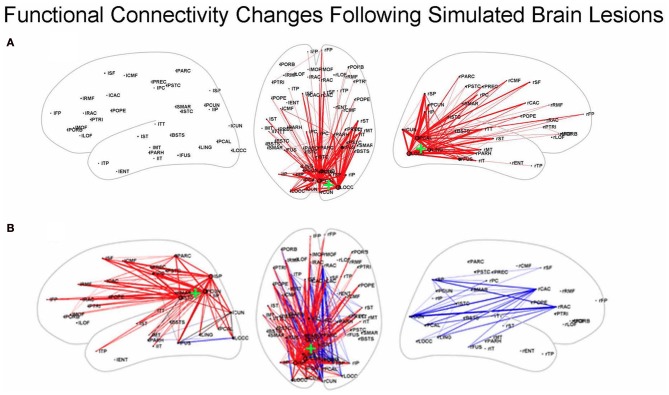
**Functional connectivity changes following simulated brain lesions (Alstott et al., 2009)**. Dorsal (middle) and left and right hemisphere views of significant changes between lesioned and normal groups in simulated resting state functional connectivity (all in the dorsal view; hemisphere specific in the lateral views) between 66 anatomical areas constructed from the 998 ROIs used by Alstott et al. Red (blue) lines indicate a decreased (increased) correlation for the lesioned brains. Center of the lesion site indicated by the green “+.” **(A)** Lesion in sensory cortex; **(B)** lesion in temporo-parietal junction. Slightly modified from Alstott et al. (2009); [**(A)** is from Supplementary. Figure 1A; **(B)** is from Figure 4B].

Other simulation studies have examined the effects of focal lesions on rsfMRI-FC, including investigations that employed structural models based on macaque connectivity (Honey and Sporns, 2008) and those that used structural data from humans (Alstott et al., 2009). We will discuss the latter of these. The structural, neural, and hemodynamic models used by Alstott et al. (2009) were the same ones as those employed by Honey et al. (2009): a DSI data set from 5 healthy human participants (Hagmann et al., 2008), the neural model of Breakspear et al. (2003) and the Friston et al. balloon model (Friston et al., 2000). A number of important findings were reported, including one showing that lesions along the cortical midline, in the temporo-parietal junction and in frontal cortex resulted in large and widely distributed reductions in rsfMRI-FC; some of these alterations involved regions outside the lesion site. In contrast, lesions of sensory and motor regions produced functional connectivity changes that were more localized to the area of the lesion (see Figure 3).

**Figure 4:**
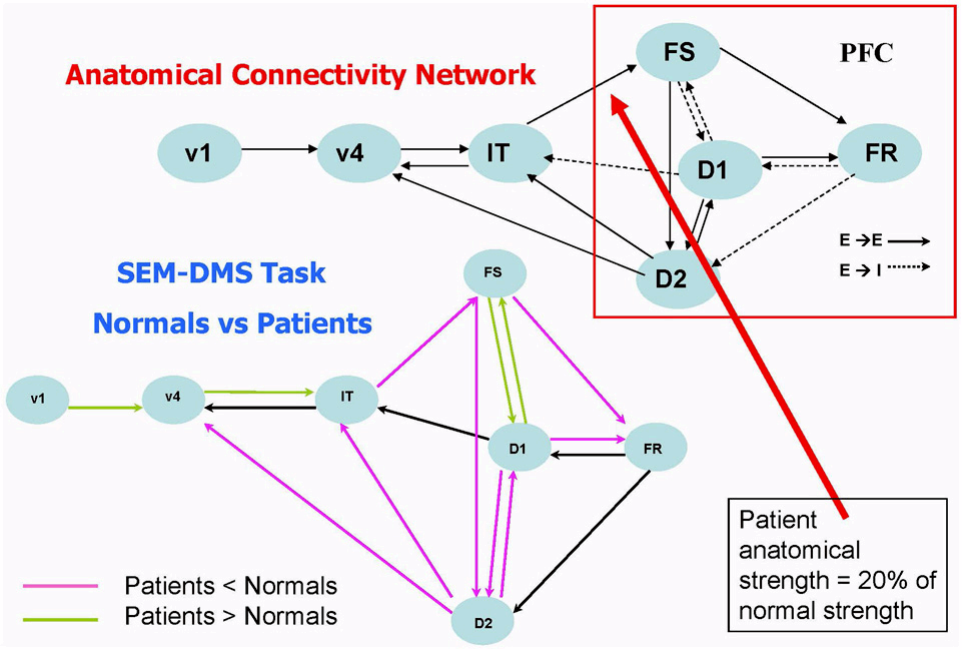
**Comparison of fMRI effective connectivity differences between simulated patients and normal subjects for a delayed match-to-sample task for visual shape (Kim and Horwitz, 2009)**. The top part of the figure shows the nodes and connections of the neural net model used (Tagamets and Horwitz, 1998) (it is the same model shown in Figure 1, which should be consulted for abbreviations). Simulated patients' data were obtained by reducing the connection weight between the IT and FS modules an average of 20% of its normal value. The lower part of the figures shows the results of applying an effective connectivity analysis (structural equation modeling) to the normal and patient networks. Significant reductions in patients relative to controls are in violet, significant increases are in green. Modified from Kim and Horwitz (2009).

The studies involving alterations in anatomical connectivity that we have so far mentioned involved simulating rsfMRI data. Task-based fMRI also has been examined using large-scale modeling, and one such paper by Kim and Horwitz (2009) investigated the effect of decreased structural connectivity on task-related effective connectivity. The general question that this study asked was: how should one interpret a significant difference between patients and controls in the effective connectivity between two nodes? In particular, does such a difference imply that there is a corresponding alteration in the underlying structural connectivity between the nodes? Kim and Horwitz used the large-scale neural model of Tagamets and Horwitz (1998), discussed in Simulated fMRI Data and Functional/Effective Connectivity, to address these questions. They reduced the strength of the structural connection from IT to PFC (see Figure 4, upper) by an average of 80% in 20 simulated “patients,” and compared the simulated fMRI obtained during the DMS task with comparable data from 20 “normal control” simulations. Structural equation modeling (SEM) (McIntosh et al., 1994) was used to evaluate effective connectivity for all the connections between all regions in the network. As shown in Figure 4 (lower), the effective connection from IT to PFC (FS) indeed was significantly reduced in the patients relative to the controls. So, this simulation result suggests that reduced structural connectivity can be reflected as reduced fMRI effective connectivity. Figure 4 also shows that the effective connectivity downstream from the induced structural disconnection (i.e., the connectivity within the PFC) also was generally reduced. This result is not unexpected: the disruption in the IT-FS connection leads to incorrect neural processing in downstream parts of the PFC network. The third result from this simulation is, at first glance, unexpected: the increased effective connectivity “upstream” (e.g., the V1–V4 effective linkage) in patients relative to controls. As mentioned in Introduction, numerous groups have reported increased patient functional/effective connectivity (e.g., for AD, Horwitz et al., 1995; Damoiseaux et al., 2012), and in many cases, this increase is attributed to some type of neural plasticity. The simulation produced by Kim and Horwitz (2009) indicates that this interpretation may not always be warranted. In the simulation, no structural alteration in the V1–V4 connections weights took place. Rather, the increased effective connectivity resulted from a reduced feedback effective connection from PFC to V4, which in turn led to V4 being more influenced by V1 activity than was the case in the normal subjects. A major conclusion from the Kim and Horwitz study was that interpretation of fMRI functional/effective connectivity changes in patients relative to controls requires a careful consideration of the entire network mediating the task under study.

What about the situation for rsfMRI-FC? Would similar findings as illustrated by the Kim–Horwitz study (Kim and Horwitz, 2009) occur, or are those interpretational problems found only in task-based fMRI studies? As Alstott et al. (2009) showed, both increases and decreases in rsfMRI-FC occurred following cortical lesions. For example, as illustrated in Figure 3B, a lesion centered in the left temporo-parietal junction resulted in strengthened rsfMRI-FC in the contralesional hemisphere. Some of these increases are due to direct loss of inputs from the lesioned area, resulting in greater functional connectivity between right hemisphere nodes. As was the case with the Kim-Horwitz example, these increases are not the result of any change in the strength of the anatomical connection weights.

The studies discussed above obviously did not consider all the complexities that are likely to be found in investigations of brain disorders. Future neural modeling efforts will be needed to address such issues as how the various kinds of neuroplasticity, which can operate over multiple time scales, even ones whose duration are within the time frame of a single scan, affect the functional/effective connectivity of relevant networks. Some of these neuroplastic changes may occur due to changes in anatomical connectivity.

It is worth noting, by the way, that we have oversimplified things by assuming that there is a clear distinction between anatomical and function/effective connectivity. At the level of neuron and synapse, however, this distinction breaks down: in which category does one place axonal sprouting and the formation of new synapses, or even the strengthening of a single synaptic contact? Indeed, one kind of connectivity change can lead to a change in the other—Hebbian learning would be an obvious example. These issues will need to be confronted in future neural modeling studies.

## The role of simulation in the development of fMRI biomarkers

An important issue that was alluded to in the Introduction was the utilization of neuroimaging for generating assorted biomarkers for brain disorders. Horwitz and Rowe (2011) have discussed the various uses for which such biomarkers could be employed^4^. These include detection or prediction of a disorder, differential diagnosis, and staging a disorder and investigating treatment efficacy.

A significant and obvious point related to biomarker development is that such markers are meant to be used on individual patients (or potential patients). As such, an important issue is how likely is it that fMRI will be able to provide sufficient signal-to-noise ratio to be usable in single subjects (Horwitz and Rowe, 2011; Damoiseaux, 2012; Vemuri et al., 2012). Most of the experimental studies we have mentioned were group studies, and although these investigations are important for discerning signal patterns that have the potential to discriminate between patients (actual or potential) and non-affected individuals (or between different types of patients), clinically useful fMRI biomarkers are still a future goal, not a present reality. Two areas of fMRI research that are likely to lead to improvements are in hardware development and in advances in the use of multivariate signal processing techniques (e.g., Smith et al., 2010); for a review, see Smith, 2012.

A second issue, implicit in our previous discussion, concerns what kind of fMRI technique (i.e., resting state fMRI or task-based fMRI) is better to use for a particular brain disorder. The answer depends on two things: which brain disorder is the focus of interest, and which question is the biomarker attempting to address. In some cases, it may be that rsfMRI will be more appropriate. For example, getting small children to do a specific set of tasks could lead to compliance problems of one sort or another. In other cases, task-based fMRI might have a significant advantage. Specifically, task-based fMRI provides the opportunity to record behavioral measures during scanning, and thus, these behavioral measures can be correlated with the changes in connectivity. This is a powerful method for determining which connectivity changes are aiding the person being scanned and which are reducing their performance. Similar behavioral correlations have been used with resting state connectivity changes, but the behavioral measure is on a subject by subject basis, not on a trial by trial basis. For example, Venkataraman et al. (2012) found two co-existing patterns of connectivity in their schizophrenia patients: increased frontal-parietal connectivity that was associated with severity of positive symptoms, and decreased parietal-temporal connectivity that was related to negative symptoms.

As an illustration of the task vs. resting issue, consider AD. We know that the pathology of AD can be found in individuals' brains decades before clinical symptoms appear (Reiman et al., 1996; Hampel et al., 2011), and young adults at risk for developing late-onset AD show default mode network (DMN) alterations (Filippini et al., 2009). Given this situation, if an appropriate therapy were available, when should it be given? One might want to start it before a patient demonstrates cognitive deficiency (in which case there may be a significant reduction in viable brain tissue), but perhaps not years or decades before, given the likely costs of the treatment and the potential side-effects of the therapy. In analogy with cardiovascular disease, a “cognitive stress test” during fMRI scanning might provide a way to assess neural integrity. However, one study (Fleisher et al., 2009) has been used to argue against task-based fMRI studies and in favor of rsfMRI in AD. Fleisher et al. showed that rsfMRI of the DMN had a larger effect size than did an fMRI encoding task for distinguishing AD high-risk from low-risk groups. However, it should be noted that although functional connectivity was utilized for the rsfMRI portion of the study, the researchers only used differences in regional BOLD deactivation in DMN nodes during the encoding part of the investigation. As Horwitz and Rowe (2011) have suggested, a task-base network analysis, targeting a network that shows early impairment in AD (such as memory), might be more sensitive compared to examining individual region of interests, since network analysis is intrinsically multivariate. One would determine if the at-risk subject's data fit the network defined by healthy control subjects performing the same task. If the fit is bad, that would suggest that therapy might be warranted. This scheme is based on the notion that neuroplasticity enables behavioral performance to be maintained during the many years during which brain pathology builds up.

As we have just seen, progress has been slow in developing fMRI based biomarkers. Among the reasons for this are the difficulty in performing neuroimaging studies on patients, and importantly, not being able to actually “know the answer.” Of the patients at risk for a given disorder, how many will actually get the disorder, and when will they get it? Patient variability is often huge, and different individuals could have different amounts of neuroplasticity over the years during which a disorder may have gone undiagnosed. How do we know that a group difference in some fMRI metric will be large enough in individuals to be able to distinguish a single subject with a high sensitivity and specificity? Note that the problem is not just scanner signal-to-noise, as was mentioned earlier. Rather, the additional problem is that there is large subject-to-subject variability in humans, even in healthy subjects—structural brain differences (e.g., see Amunts and Zilles, 2001), and functional differences (e.g., see Kanwisher and Yovel, 2006).

Computational neural modeling may provide a method to circumvent some of these issues in attempting to determine if an fMRI based metric can serve as a biomarker for detecting a brain abnormality. As an illustration, how weak can a brain structural disconnection be so that it is undetectable using rsfMRI-FC analysis? In our review of the simulation studies of Deco, Cabral and their group and Alstott, Honey, Sporns and their colleagues, the extent of the structural damage was quite large in many cases. For example, Alstott et al. (2009) found in one of their analyses many significant differences in functional connectivity in 5 subjects when they deleted 50 ROIs from an anatomical area (see Figure 3 for two examples). Using the same set of models (structural, neural, and hemodynamic) as Alstott and collaborators, we targeted two anatomical areas for modification: the left precuneus (LPr) and the left medial frontal cortex (LMPF). All modifications were performed on the 25 ROIs closest in Euclidean distance to the center of the targeted areas. Specifically, the structural connectivities in a targeted area were scaled by 0.5 from the normal values. We examined focal, unidirectional, and bidirectional modifications. In focal alterations, connections among the 25 ROIs in a single anatomical area were scaled by 0.5, but connections between these targeted ROIs and all other ROIs in the cortex were left unmodified. Bidirectional and unidirectional structural alterations were only applied to the two separate anatomical regions—LPr and LMPF. In bidirectional modifications, the connections from one set of 25 target ROIs to and from the second set of 25 target ROIs were scaled by the specified amount of 0.5. In unidirectional modifications, the connections from one set of ROIs in LMPF to the LPr set of ROIs were scaled, but the connections from the latter set of ROIs to the former set were left intact.

Simulations were run for 10 “normals” subjects and 10 “patients.” Variation in the subjects was introduced by adding or subtracting to all the structural connection weights random numbers from a Gaussian distribution with a standard deviation of 0.01. Pearson correlations between the time series of the simulated BOLD activity from each anatomical area for each “normal” subject and for each “patient” were evaluated. Given the small number of “subjects” (10 in each group), and the relatively weak reduction of structural connectivity between just two brain areas, it is not surprising that there were few robust group differences. Indeed, no significant group differences in rsfMRI-FC between the two targeted areas LPr and LMPF were found in any of the cases (focal, unidirectional, bidirectional). These simulation results thus indicate the relative insensitivity of simple rsfMRI-FC to detecting the presence of structural modifications that are weak and of restricted extent, even if one knows where to look. That is, not much change occurs when one input is reduced to areas that have inputs from multiple other areas. Simulations could be used to see if the situation is different when the modification affects a connection between nodes engaged in a task, as was the case for the Kim-Horwitz simulation (Kim and Horwitz, 2009) that was discussed earlier, but that would require adjusting the structural and neural models so that a specific task can be performed. Moreover, newer experimental and data analysis procedure could arise to improve the situation. For instance, high spatial resolution MRI may be able to find mild abnormalities in either structural or functional connectivity in the future.

## Conclusions

In this paper we reviewed some recent efforts at using neural modeling to help understand and interpret human neuroimaging data comparing patients with brain disorders to healthy subjects. Experimental neuroimaging data provide macroscopic measures of brain structure and function. In the case of fMRI, these data are indirect measures of function; the signals are those of the metabolic/hemodynamic consequences of neural activity. Among the factors confounding the interpretation of such data in patients are the sheer complexity of neural anatomy and connectivity and the immense plasticity of the brain. Large-scale neural modeling provides a way to study such a system and investigate how the size and extent of various modifications translate into alterations in neuroimaging signals. Furthermore, because we know what alterations actually took place in the modeled brains, potential interpretations of actual data can be checked against the simulated data.

Our review of several studies that explored the fMRI consequences of alterations in anatomical connectivity lead to several conclusions. First, interpretation of changes in either functional or effective connectivity is not as straightforward as one might first suppose. Although a weakening of the structural connection strength between brain areas can appear as a decreased functional/effective connection, decreases and increases in functional/effective connectivity between areas not directly affected by the brain alteration are also found. Essentially, one must keep in mind that in a functional network, one cannot just change one link; functional networks are such that changes in one part of the network result in changes everywhere else (although not all these changes will be large enough to be statistically significant). Moreover, some of the changes in parts of a network unaffected by the structural alterations may result in a strengthening of the functional/effective connectivity, but these changes are not necessarily the result of neuroplasticity. Task-based fMRI may be a better choice than rsfMRI to deal with this issue, since it is often possible in task-based fMRI to acquire performance data during the scanning. Such data can then be correlated with the measured functional/effective connectivity, and the results of such an analysis may strengthen a claim for neuroplasticity mediating the altered connection. The net conclusion from all this is that the reverse inference—that a change in functional/effective connectivity in a patient means that there is a corresponding change in the underlying structural connectivity—is unwarranted.

We also discussed utilizing large-scale neural modeling as a tool for helping to develop fMRI resting state and/or task-based biomarkers for brain disorders. This is an area that is just beginning, but it does have potential advantages, especially in terms of cost and time. It is cheaper and less time consuming to run a large number of simulations than it is to find subjects and run fMRI experiments. But little work has been done in this area, so it will be a while before one can assess whether or not modeling can provide significant help in deciding which potential biomarkers are viable.

### Conflict of interest statement

The authors declare that the research was conducted in the absence of any commercial or financial relationships that could be construed as a potential conflict of interest.

## Footnotes

^1^Please note that we use the term “brain disorder” quite loosely. In particular, because there are both structural and functional changes during normal development and normal aging, studies of either of these processes can be considered here, since many of the issues that come about when comparing patients and healthy subjects would also be present when comparing subjects in different age groups.

^2^See Appendix for a list of all abbreviations used in this paper.

^3^The two matrices shown in Figure 2 were generated by the current authors using the structural, neural, and hemodynamic models originally employed by Honey et al. (2009) and Alstott et al. (2009).

^4^The Horwitz-Rowe article focused on neurodegenerative disorders; however, many of the points made are relevant for numerous brain disorders.

## Appendix

### Abbreviations:

AD: Alzheimer's Disease
BOLD: Blood oxygenation level dependent
DMN: Default mode network
DSI: Diffusion spectrum imaging
DTI: Diffusion tensor imaging
fMRI: Functional magnetic resonance imaging
LPr: Left precuneus area
LMPF: Left medial prefrontal area
PET: Positron emission tomography
PFC: Prefrontal cortex
rCBF: Regional cerebral blood flow
ROI: Region of interest
rsfMRI: Resting state fMRI
rsfMRI-FC: Resting state fMRI functional connectivity
SEM: Structural equation modeling
TR: Repetition time
